# A Preliminary Evaluation of the Public Risk Perception Related to the COVID-19 Health Emergency in Italy

**DOI:** 10.3390/ijerph17093024

**Published:** 2020-04-27

**Authors:** Giulia Motta Zanin, Eleonora Gentile, Alessandro Parisi, Danilo Spasiano

**Affiliations:** 1 Department of Civil, Environmental, Land, Building Engineering and Chemistry (DICATECh), Polytechnic University of Bari, 70100 Bari, Italy; giulia.mottazanin@poliba.it (G.M.Z.); danilo.spasiano@poliba.it (D.S.); 2 Department of Basic Medical Sciences, Neuroscience and Sense Organs (SMBNOS), University of Bari “Aldo Moro”, 70100 Bari, Italy; eleonora.gentile@uniba.it

**Keywords:** risk management, SARS-CoV-2, survey, biological hazard, decision-making

## Abstract

Governments faced with the spread of COVID-19 pandemic are adopting strict and severe mitigation measures to influence people’s behaviors. Public perception of health risk plays a key role in the adoption of these actions, in people’s feelings, and in their daily habits. To support decision makers from international to local levels to face with future sanitary emergencies, this study aims at investigating Italian public perceptions of health risk. To this purpose, a questionnaire was designed and administered within the period of Italian COVID-19 lockdown and quarantine to almost 9000 citizens in Italy and abroad. The obtained results highlight a significative influence that mass media play on both the level of knowledge and the feelings of the respondents. The findings also point out future variations of some perceived behaviors consequent to the COVID-19 outbreak.

## 1. Introduction

The novel coronavirus disease (COVID-19), caused by the virus named SARS-CoV-2 (previously 2019-nCoV), is a highly infectious disease [1,2,3] and, due to the rapid increase in the number of cases from December 2019, it was classified by the World Health Organization (WHO) as a pandemic on 11 March 2020 [4].

Specifically, in late December 2019, Chinese doctors highlighted clusters of patients with pneumonia of unknown cause, epidemiologically linked to a wholesale market in Wuhan, Hubei Province [5,6,7]. The situation became so critical that, on December 31, 2019, the Chinese Center for Disease Control and Prevention promoted an epidemiologic investigation on this new disease. As a result, both the Chinese public health and international scientific communities began to work on this topic and quickly recognized a new coronavirus, sharing the viral gene sequence with the world [3,8]. Meanwhile, despite the security and mitigation measures taken by the Chinese government, including quarantine in Hubei Province, infections spread across China and, today, it has affected more than 200 countries worldwide [9,10]. Consequently, on 30 January 2020, the WHO declared this outbreak a Public Health Emergency of International Concern (PHEIC) [4]. According to the International Health Regulations [11], a PHEIC occurs when there is an unusual or unexpected event with a serious health impact, a significant risk of international spread, and a significant risk of restrictions on international trade or traffic. 

Thus, SARS-CoV-2 is primarily affecting a common good, that is public health. Its spread is resulting in a public health disaster that is evolving in a cascading way because of the strong and high interdependency between most countries from political, economic, and societal points of view [12]. As a matter of fact, in a strongly interconnected world, several negative consequences are happening beyond the health systems from local to international scales, e.g., the interruption of production, and consequent negative consequences on global financial markets and the tourism industry [13,14]. 

The public perception of biological hazard plays a key role in the response to health emergencies, affecting risk management and risk communication strategies [15,16]. Therefore, the public perception of health risks can influence markets, public policies, and individual behaviors [17].

In the last decades, many countries have been implemented policies to cut public spending. As a consequence, despite the United Nations have stressed the necessity to strengthen health resilience [18], the investment in public health systems have decreased [19,20], increasing vulnerability and exacerbating the negative effects of this pandemic [21].

Another factor affecting the rapid worldwide spread of the SARS-CoV-2 is related to the difficulty in detecting infected people because of the lack of symptoms, as well as to similarities with symptoms of common cold and flu [7,22]. Consequently, as already happened during the first coronavirus spreading [23], it gives the opportunity to infected people to travel a significant distance from the infection site, transporting the biological agent and potentially spreading the virus in uncontaminated areas [24]. 

As reported during other similar health emergencies [24], decision makers at international, national, regional, and local levels should implement strict and unpopular public health measures to prevent and reduce the biological risk consequent to the virus spread, such as lockdown and quarantine. Beyond being restrictive measures that limit displacement and gatherings, social distancing contributes to divide many families and groups of friends [25,26]. These measures are focused on slowing the outbreak spread and reducing the peak healthcare demand, with the scope of flattening the infection curve and reducing the peak of the outbreak [27,28]. Moreover, these actions attempt to protect, as in this case, those people who are most at risk of severe disease from infection, in particular those with chronic health conditions and older people [29,30]. However, the radical change in daily habits, the limitation of social life and the stress resulting from the public-health emergency could have a strong impact on the well-being of individuals [31,32,33].

As highlighted by the WHO during the spreading of the first coronavirus [34], the SARS-CoV-2 emergency represents a major global public health threat which requires a coordinated global response. However, there is a general interest towards the promotion of public health measures at individual and local scales to prevent disease rather than common public health actions [35,36]. In particular, the lack of coordinated responses among the governments of countries involved in this public-health emergency has been observed both in terms of response time and adopted actions. This might be due to the creeping nature of this kind of hazard, which begins to concern only when the negative effects of the trigger factor become visible and tangible and the emergency could already have reached the critical transition, shifting in a cascading disaster [12,37]. As an example of the uncoordinated responses, even if the Chinese government applied draconian mitigation measures in the worst-affected areas from 23 January 2020, in the EU, UK, and USA, it was necessary to wait at least one month to see similar containment measures applied. Therefore, although in China few or no cases of internal infections caused by SARS-CoV-2 have been currently registered, other countries are still faced with the virus spreading and its negative effects.

Generally, some cross-cutting aspects, such as communication, stakeholder engagement and context, are fundamental in order to cope with hazards and risks in situation of high complexity, uncertainty, and ambiguity [38,39]. As argued by Slovic [16,17], those who assess and manage public health and safety should deeply investigate the way in which people perceive and face with risks. As a matter of fact, most of people who face hazardous phenomena rely on intuitive risk evaluation, so called risk perception, which is unavoidably influenced by both mass media and social contacts with friends, relatives, and colleagues [40]. With regard to the influence of mass media, it is important to stress their role in public perception of health risk, inasmuch they often give information mainly focused on mishaps and threats occurring in affected countries [16]. Since people usually make decisions based on their risk perception rather than the effective risk [15], public perception of health risk plays a key role in the adoption of measures, in their acceptance, in the feelings of the population, and in the decisions that people will take. 

During a public-health emergency, measures taken by governments, such as lockdown and quarantine, may heavily interfere with the individual choices of citizens, varying their daily habits and behaviors. Thus, decision makers, who are usually prone to act according to the effective risk [41], should consider public perception of health risk, as well as its communication for an effective emergency and risk management [38]. Moreover, risk management and communication need to be structured considering both public perception of health risk and experts contributions as a two-way process, otherwise efforts to manage public emergencies risk to fail [16].

In particular, the public underestimation of health risk might reduce the acceptance of the strict mitigation measures enforced by governments [42]. On the contrary, some feelings, such as fear and anxiety, are more likely to cause overestimation of the health risk [43,44]. Notably, previous studies suggest that the spread of a virus can contribute to a widespread sense of panic and concern in the community [44,45]. The perception of the level of lethality of a virus seems, in fact, to be associated with the development of emotional distress [45,46], which involves the need to structure psychological assistance interventions [47]. Moreover, at list in the short term, social media may affect feelings and, consequently, risk perception [48]. Consequently, the information provided by the media and other official sources, as well as the way in which they are communicated, play a key role during these emergencies [49]. By dealing with the Italian case study, this work aims at providing preliminary insights, which might support decision makers to face with future sanitary emergencies, by investigating public perception of health risk. As already stressed, it represents a fundamental aspect to better comprehend and define the most effective strategies, as well as to make mitigation measures as acceptable as possible in countries affected by this kind of emergency.

In order to provide these insights, the public perception of health risk has been investigated through the administration of a questionnaire to Italians living in Italy (ILI) and Italians living abroad (ILA) within the period of lockdown and quarantine in Italy. In addition, some aspect have been analyzed and compared, such as the level of acceptance of the mitigation measures taken by Italian Government, the level of knowledge regarding the virus widespread in Italy, and some effects of this public health emergency to the feelings, concerns, and behaviors of ILI and ILA.

## 2. Case Study 

As in the rest of the world, the first information regarding the COVID-19 arrived in Italy in December 2019. Later, the formalization by the WHO about the appearance of a new coronavirus (7 January 2020), the lockdown in Wuhan and other cities in Hubei province (23 January 2020), the statements by Chinese President Xi regarding the acceleration of the coronavirus spread (25 January 2020), and, finally, the rising in the level of risk by the WHO (26 January 2020) increasingly caught the attention of the Italian Government and public opinion. 

On 23 January 2020, a couple of Chinese tourists arrived in Milan from where they had moved for a tour in the Italian provinces before arriving in Rome. Unfortunately, on 30 January 2020, they showed relevant symptoms of COVID-19 and were admitted to the “Lazzaro Spallanzani” National Institute for Infectious Diseases (Rome), where the positivity for SARS-CoV-2 was confirmed. This represents the first case of SARS-CoV-2 transmission described in Italy and prompted the Italian Government to declare the state of emergency [50]. During the same day, the WHO declared this outbreak a Public Health Emergency of International Concern (PHEIC). 

Some days later, on 6 February 2020, an Italian citizen repatriated in Rome from China was tested positive for SARS-CoV-2 and quickly hospitalized: he represents the first Italian patient. Instead, the first Italian who contracted the SARS-CoV-2 in Italy was a 38-year-old man admitted to the hospital of Codogno (Lodi, Lombardy, Northern Italy), a town of almost 16,000 inhabitants, on 20 February 2020. During the following 24 h, another 15 infected were found in the Lombardy region and another outbreak of infection was discovered in Vo’, a small village of almost 3000 inhabitants in Padua province (Veneto, Northern Italy). In the same day, in Padua, the first death was registered. He was a 78-year-old man, unfortunately the first of a long series.

On 23 February 2020, to curb the spread of the virus, the Italian Government took the first special measure limited to the outbreak areas. Specifically, in 10 municipalities of Lodi province and in the town of Vo’ began the ban on exiting and entering with penalties for those who violate the regulations, the interruption to school trips in Italy and abroad, and the closure of schools, shops and museums. On 4 March 2020, the number of deaths caused by SARS-CoV-2 rose to over 100 and the Government imposed the closure of schools and universities in the whole country. However, the number of infected and deaths continued to increase, and, in the night between 7 and 8 March 2020, the leak of news about another decree (not yet approved), aimed at prohibiting any movement in Lombardy region and in fourteen provinces of Veneto, Emilia Romagna, Piedmont, and Marche, wreaked havoc across the country. On that night, there was an ‘escape’ of thousands of people from several cities of Northern Italy to the south. As an example, more than 400 people, most of whom are Italian people coming from the south and living in Northern Italy seasonally mainly for study purposes, reached the Milan train station to take the last train to Southern Italy. This exodus seriously endangered people in the south, where the virus had not yet revealed its destructive power. Consequently, on the night of 9 March 2020, the Prime Minister Giuseppe Conte, with the decree called #IoRestoaCasa (#istayathome), proclaimed a state of emergency across the whole country, allowing the exit from homes only for necessary work activities, the purchase of basic necessities, or for health reasons. One of the measures to reduce SARS-CoV-2 infection taken by the National Government, in agreement with the Civil Protection Department and the Higher Institute of Health, was the definition of a minimum interpersonal distance of one meter [51].

**Figure 1:**
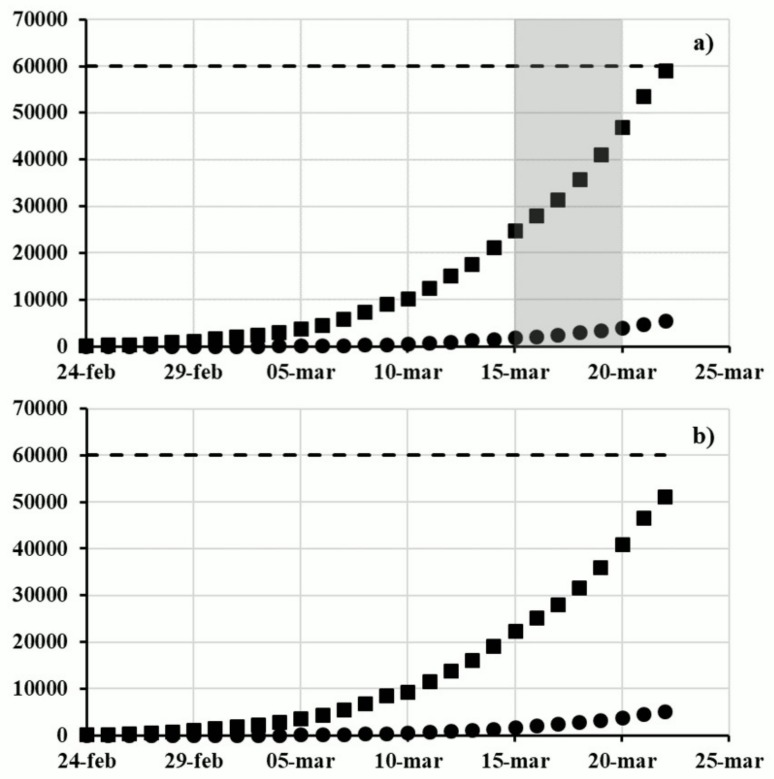
Cumulative trend of the number of infected people (■) and death (●) in (**a**) Italy and in (**b**) the more affected Italian regions. Dashed line represents the 0.1% of the Italian population. The highlighted area represents the period of the questionnaire administration (Data from the Italian Civil Protection Department: https://github.com/pcm-dpc/COVID-19—last access: April 27, 2020).

Despite the imposition of these security measures, the number of infected people and deaths continued to grow. Specifically, Figure 1a shows the numbers of infected people and deaths with SARS-CoV-2 daily communicated by the Italian Civil Protection Department. These official data are very important since their update was communicated every day at 6:00 pm by the main media (social media and television). In particular, the number of infected people was lower than 0.1% of Italian inhabitants until 20 March 2020 (see the dashed line in Figure 1) and the lethality, expressed as the ratio between the deaths and the infected people, was in the range of 7.3–8.3%. 

**Figure 2:**
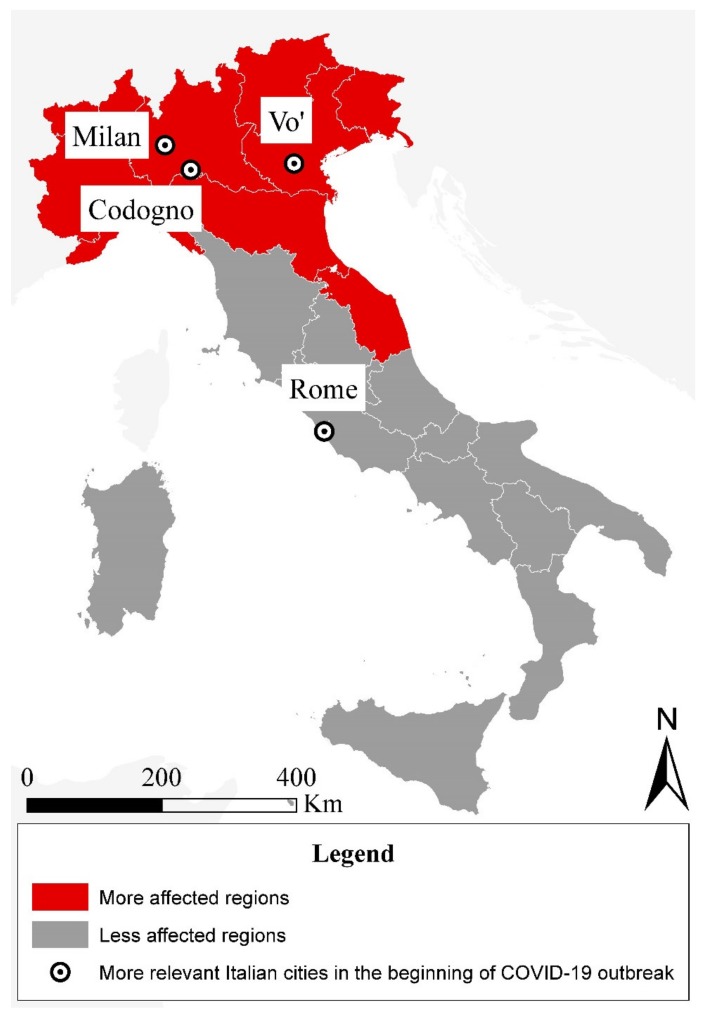
Framing the more affected Italian regions (red) and less affected ones (dark grey) by the spread of the SARS-CoV-2 (up to the 15 March 2020).

Until that date, the number of infected people and deaths also increased in the regions bordering those previously reported (Figure 2). Specifically, as early as 15 March 2020, the Italian regions where the death toll caused by the spread of SARS-CoV-2 corresponded to more than 10 people per million inhabitants were Lombardy, Emilia Romagna, Marche, Liguria, Piedmont, Veneto, Friuli Venezia Giulia, Trentino Alto Adige, and Valle D’Aosta. Moreover, the number of infected and dead people in these more affected regions represented almost the whole number of positives and victims at the national scale (Figure 1b).

## 3. Data and Methods

### 3.1. Participants and Procedure

Public Italian perception of health risk was investigated through the administration of a questionnaire, designed, and structured in seven blocks described hereafter. The data collection was carried out on the sample of the Italian population living in Italy (ILI) and abroad (ILA). The questionnaire was administered via e-mail, through mobile phones and social networks at the beginning of the Italian lockdown and quarantine (from 15 March to 20 March 2020). A stratified random sampling method was chosen to select the respondents and to ensure the representativeness of the whole Italian population. Moreover, this method allowed all people within the target population an equal chance of being selected [52].

### 3.2. Questionnaire Structure

The questionnaire was designed using closed-ended questions, except in a few specific cases. The items were sorted to allow a gradual transition from one topic to another [53] and are summarized as follows:

1. Introduction to the study, invitation to participate and demographic information

First, the aims of the research were illustrated, and respondents were asked to express their consent to participate. The questionnaire began with basic demographic information related to sex, age, level of education, and job status.

2. Information regarding the living place and social contacts of respondents

In this section, information about country, region/province, number of inhabitants, and number of daily social contacts of interviewees were asked.

3. Assessment of the perceived general health status and susceptibility perception of COVID-19

In order to investigate some general aspects perceived by respondents, a question focused on the quality of their own health status was submitted. This item derived from the SF-36 Health Surveys [54] adapted for the Italian population [55]. In this case, response options were excellent, very good, good, fair and poor. Moreover, interviewees were asked about their perception of susceptibility with respect to COVID-19. In particular, the possible answers were: no, yes, due to my age and yes, due to my health status. Finally, the following question was administered: “Have you ever been in direct contact with a person affected by SARS-Cov-2?”. In this case, the response options were: yes, no, I don’t know, and I prefer to do not answer.

4. Experienced feelings

In this section, respondents were asked to describe their actual feelings about the Italian sanitarian emergency state. Specifically, the possible choices were: nothing in particular, uncertainty, sadness, fear, anger, and other. In the latter case, participants had to specify their feelings answering an open question.

5. Background information about the spread of SARS-CoV-2 in Italy

Participants were asked to indicate the main sources of information adopted to inquire news regarding the spread of COVID-19 outbreak among television, radio, newspapers, scientific journals, social networks, general practitioner, relatives, and friends. Moreover, it was asked to express their level of trust on the information received by National Government, Italian Civil Protection Department, government at regional and local level, general practitioner, relatives and friends, and mass media. In particular, the response options were: none, few, neither few nor much, much and very much. Later on, the following questions regarded the period in which the first SARS-CoV-2 positive test in Italy was registered (December 2019; January 2020; February 2020; March 2020), the place where the first positive test was recorded (Rome; Codogno; Milan; Vo’), the ratio between the number of infected people and the Italian population (<0.1%; 0.1–0.5%; 0.5–1%; 1–10%; >10%), and the lethality of COVID-19 in Italy (<0.1%; 0.1–0.5%; 0.5–1%; 1–10%; >10%).

6. Perceived efficacy of the mitigation measures imposed by the Italian Government

In this section, respondents were asked if the action taken by the Italian Government about the public health emergency were adequate or not (response options: yes; no; I don’t know). Those who answered “no” were asked to specify the reason of their disagreement (response options: late measures were taken; there was no clear communication; the measures were not stringent enough; government actions were disorganized; the measures were too stringent; other). For ILA, an additional question regarding their perception about the comparison between the actions adopted by the Italian Government compared to ones adopted (or not) by the countries where they live was provided (response options were ranged from 1 = much worse to 5 = much better).

7. Perceptions of the foreseen effects of the COVID-19

In this section, participants answered to the following question: “how do you think COVID-19 in Italy will affect the following aspects?”. In particular, the investigated aspects were environment, culture, demography, economy, politics, and social. The possible choices were: I don’t know, much worse, worse, not affected, better, and much better. Later, the respondents were asked to foresee the end of the Italian public health emergency (within the end of April 2020, within the end of May 2020, within the end of June 2020, within the end of September 2020, within the end of December 2020, after December 2020), the ratio between infected people and the Italian population at the end of the Italian public health emergency (<0.1%; 0.1–0.5%; 0.5–1%; 1–10%; >10%), and the lethality of COVID-19 in Italy at the end of the Italian public health emergency (<0.1%; 0.1–0.5%; 0.5–1%; 1–10%; >10%). The last question was about the expected changes in the interpersonal distances. As described by Edward Hall [56,57], they are intimate distance (<0.45 m), personal distance for interaction between friends (0.45–1.2 m), social distance for communication between acquaintances (1.2–3.5 m), public distance (>3.5 m). The response options regarding the changes were: very reduced, slightly reduced, no changes, slightly increased, and very increased.

### 3.3. Data Analysis

The statistical data analysis was performed using IBM Statistical Package for Social Sciences (SPSS) Statistics software, version 21. First of all, respondents that gave invalid answers, such as Italians who declared to live abroad and gave a non-existent country of provenience, were excluded from the analysis (*n* < 0.2%). Thus, the analyzed sample was constituted by 8282 Italians living in Italy (ILI) and 431 Italians living abroad (ILA) (Table 1). The age range of both ILI and ILA was 18–80, whereas the mean age of ILI was 40 years (SD = 14) and the mean age of ILA was 35 years (SD = 11). 

ILA responded from all five continents and, precisely, from 42 countries worldwide. ILI answered from all the Italian regions and provinces, although with different percentage of response. In order to avoid the overall result being affected by the regions with the most respondents, it was decided to weight the answers dividing the responding sample. Aware of the different situations experienced within the Italian territory during the administration period of the questionnaire, the sample of ILI was divided into two sub-clusters considering Italian living in Italy in more affected regions (ILI-MAR) and in less affect regions (ILI-LAR). In detail, the more affected Italian regions were considered the ones with a death toll caused by the spread of SARS-COV-2 corresponding to more than 10 people per million inhabitants (see Section 2 and Figure 2). Finally, the result of ILI was obtained through a weighted average of the two sub-clusters. 

In order to evaluate the reliability of analyzed sample of respondents, the Cochran formula was applied [58,59]. As shown in Table 2, all the chosen clusters of respondents have more than 400 interviewees and, as highlighted by Kellens et al. [60], studies with cluster of respondents higher than 400 are considered statistically significant. According to the size of the corresponding samples, the calculated *p*-value results less, at the most equal, to 0.05. In detail, the *p*-value assessed for both ILI-MAR and ILI-LAR is 0.02, the former with a confidence level of 95%, the latter with a confidence level of 99%. Consequently, *p*-value of ILI is 0.02 with a confidence level of 95%, whereas *p*-value assessed for ILA is 0.05 with a confidence level of 95%. 

In order to analyze the results of the questionnaire, a descriptive statistical analysis was carried out. Notably, not all the answers were analyzed and discussed in this work inasmuch some of them are related to future aspects. Thus, their analysis will be performed when the Italian public health emergency will be ended.

## 4. Results and Discussion

The results and related discussions reported in this section regard the data collected through a questionnaire submitted from 15 March to 20 March 2020 to ILI and ILA. Moreover, a further analysis of the results distinguishing ILI-MAR and ILI-LAR was carried out in order to highlight possible differences in risk perceptions related to COVID-19 in Italy.

### 4.1. Assessment of the Perceived General Health Status and Susceptibility Perception of COVID-19

**Figure 3:**
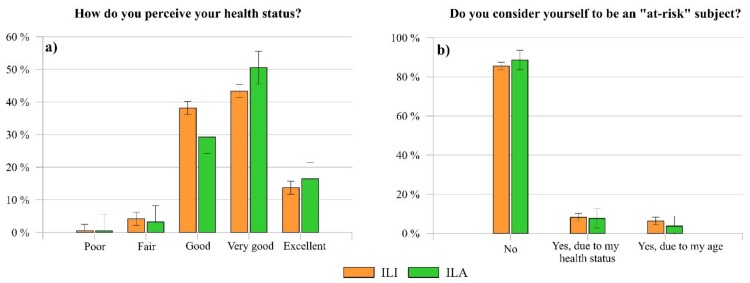
Comparison between the answers of ILI and ILA regarding (**a**) their perceived health status and (**b**) the susceptibility perception of COVID-19.

As far as the assessment of the perceived health status of each responder is concerned (Figure 3a), most of them reported that they have a good (ILI 38.19%, ILA 29.23%), very good (ILI 43.38%, ILA 50.58%), or excellent (ILI 13.74%, ILA 16.47%) health status. Instead, a smaller percentage of the sample stated a poor (ILI 4.16%, ILA 3.25%) and a fair (ILI 0.53%, ILA 0.46%) health status. Further, more than 80% of ILI and ILA believes to be not at-risk of SARS-CoV-2 infection (Figure 3b).

This result is consistent with the general subjective assessment of the participants health perception. Indeed, most of the respondents, who declared a positive health status, also stated not to be at-risk except for the age. However, it is worthy to observe that almost 8% of participants reported to be at-risk due to their health conditions, even if almost 4% declared to have an either poor or fair health status. Analyzing the perceived health status of the people who consider themselves at-risk due to the health conditions, almost 60% of them declared to have a good or very good health status. This discrepancy might be due to the presence of mild disorders or non-disabling clinical conditions that do not significantly affect the perception of one’s health status, but which could play a role in the perception of one’s susceptibility to a possible SARS-CoV-2 infection.

### 4.2. Experienced Feelings

**Figure 4:**
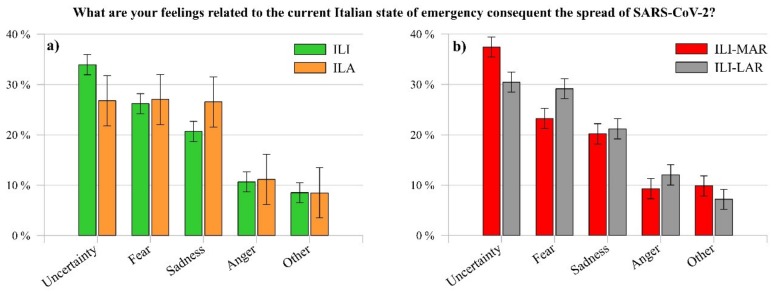
Comparison between the feelings experienced by (**a**) ILI and ILA, and by (**b**) ILI-MAR and ILI-LAR.

Regarding the question “What are your feelings related to the current Italian state of emergency consequent the spread of SARS-CoV-2?”, the participants mainly expressed uncertainty, fear, and sadness (Figure 4).

Whilst the feeling of fear experienced by ILI (26.20%) and ILA (27.03%) results similar, the feelings of sadness (ILI 20.68%, ILA 26.54%) and uncertainty (ILI 33.94%, ILA 26.79%) show a relevant difference. The rapid spread of the outbreak in Italy has presumably elicited a general state of uncertainty in ILI, who suddenly face with a little-known threat [61]. Moreover, since Italy was one of the foremost countries in Europe and in the world severely facing the spread of the pandemic, and that it imposed a lockdown and quarantine on its inhabitants, ILI result is more uncertain than ILA, who did not directly face strict health mitigation measures. As a consequence, ILA result sadder, probably because of the critical situation that ILI, in particular relatives and friends, were experiencing in Italy. 

Although Figure 4a shows that the prevailing feeling among ILI is uncertainty, Figure 4b reports a notable difference between the same feeling experienced by ILI-MAR (37.43%) and ILI-LAR (30.46%). Probably, the former negative effects of mitigation measures on the production system (e.g., closed factories, suspended or cancelled orders and layoffs) might have generated a higher uncertainty feeling in ILI-MAR. As the matter of fact, they live and work in the regions with the higher Italian GDP (Gross Domestic Product), corresponding to the 58% of the Italian GDP [62]. Indeed, in line with this consideration, a recent study conducted in the USA reported that the economic uncertainty induced by the spread of the virus could reflect a general feeling of uncertainty in the population [63].

To whom concern the feeling of sadness, it does not show a relevant difference (ILI-MAR 20.19%, ILI-LAR 21.18%), whereas fear is higher in ILI-LAR (29.15%) than in ILI-MAR (23.24%).

The different feelings expressed by them might be a consequence of the distinct situation that the population of the two Italian areas (the more affected regions and less affected ones) was living. On the one hand, at the end of February 2020, ILI-MAR started to face with the critical negative consequences of the SARS-CoV-2 spread and restrictions of the Italian government. On the other hand, at that time, ILI-LAR were just observing “from outside” the situation in the more affected regions when, suddenly, the lockdown and quarantine were declared in the whole country (9 March 2020). Probably, the looming threat of the virus spread, dramatically described by mass media and the Italian government regarding the increasing number of positive cases and deaths, may have contributed to a greater expression of fear in ILI-LAR than in ILI-MAR. 

It is worthy to report that anger was chosen for 9.30% of ILI-MAR and for 12.04% of ILI-RAR, whereas 8% of participants chose the answer other feelings and the most indicated feelings were worry (ILI-MAR 1.18%, ILI-LAR 72%), hope (ILI-MAR 68%, ILI-LAR 38%) and anxiety (ILI-MAR 60%, ILI-LAR 50%).

### 4.3. Level of ILI and ILA Knowledge about the Spread of SARS-CoV-2 in Italy

**Figure 5:**
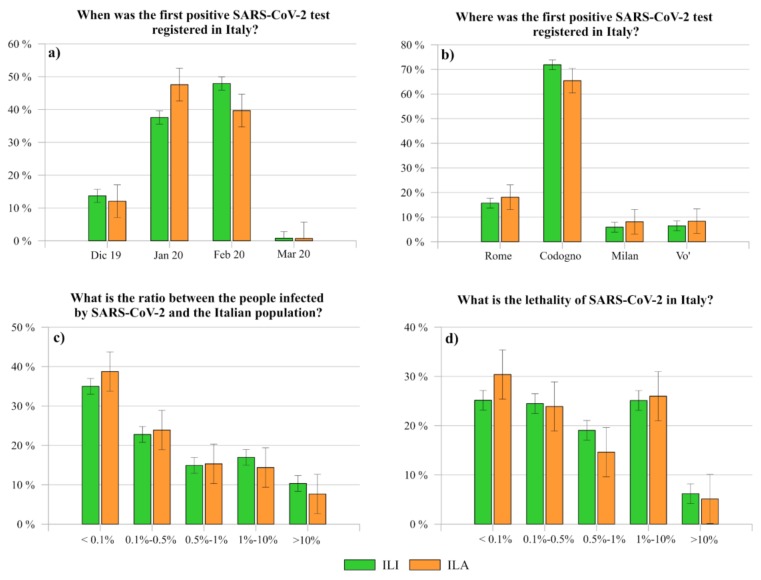
Comparison between the level of knowledge of ILI and ILA about the date of the first positive SARS-CoV-2 test registered in (**a**) Italy, (**b**) the place where it was registered, (**c**) the ratio between the number of people infected by SARS-CoV-2 and the Italian population, and (**d**) its lethality in Italy.

As reported in the second section, the first positive SARS-CoV-2 test registered in Italy was in Rome to two Chinese tourists at the end of January 2020. As shown in Figure 5a,b, when respondents were questioned “When was the first positive SARS-CoV-2 registered in Italy?”, 37.57% of ILI and 47.56% of ILA answered correctly, while 47.92% of ILI and 39.68% of ILA thought that February 2020 was the correct one (Figure 5a). As it is possible to see, ILA seems to have a better knowledge than ILI about this information. However, with respect to the question “Where was the first positive SARS-CoV-2 registered in Italy?”, less than 20% of both respondents gave the correct answer, while more than 60% answered with Codogno (Figure 5b). These results highlight that, on the one hand, the information about the positive SARS-CoV-2 test of the two Chinese tourists at the end of January was probably either neglected or forgotten by the interviewees. On the other hand, the media bombing regarding the first case of an Italian infected in Codogno at the end of February 2020, as well as the following daily updates regarding the increasingly critical situation of that small town in northern Italy, might have contributed to the wrong awareness of the Italian citizens in Italy and abroad. 

With regard to the number of infected people with respect to the Italian population (officially, <0.1% up to 20 March 2020) and to the lethality of COVID-19 (officially, 7.3–8.3%, up to 20 March 2020), Figure 5c,d shows the knowledge level of the interviewees. 

In particular, even if no significative differences were registered between the two clusters of participants, also in these cases a remarkable confusion is reported. Specifically, less than 40% of participants were demonstrated to be aligned with the official data and only 25% knew the correct ratio of disease lethality. As regard to the number of infected people, it is worthy to report the discrepancies between ILI-MAR and ILI-LAR. In particular, 39.14% of the former declared that the ratio between infected people and Italian population was less than 0.1% against the 30.85% of ILI-LAR, who demonstrated a overestimated perception of the outbreak spread with respect to the official data. However, the real amount of Italian infected people and, consequently, the lethality is still debated since SARS-CoV-2 tests were mainly carried out just on patients who showed remarkable symptoms of COVID-19 disease. Consequently, the official data provided by the Italian Civil Protection Department does not considers asymptomatic and the infected people who have developed only mild symptoms. As a result, it is understandable that incomplete data might cause further confusion within the population.

### 4.4. Main Sources of Information Related to the Spread of SARS-CoV-2 in Italy

**Figure 6:**
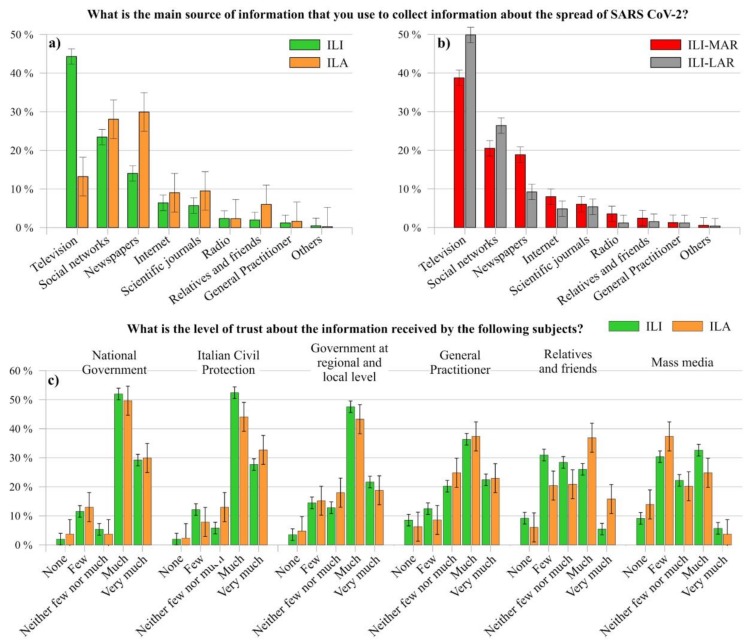
Comparison among ILI and ILA (**a**), and among ILI-MAR and ILI-LAR (**b**), as regard to their main source of information about the spread of SARS-CoV-2; comparison among ILI and ILA (**c**) as regard to their level of trust of the information received by the above-cited subjects.

As shown in Figure 6a,b, the main source of information related to COVID-19 in Italy adopted by ILI is the television (44.31%), followed by social networks (23.45%) and newspapers (14.04%). On the contrary, ILA firstly refer to newspapers (29.93%), secondly to social networks (28.07%) and finally to television (13.23%).

Some distinctions emerge also between ILI-LAR and ILI-MAR. As a matter of fact, there is a difference of around 10% regarding people who mainly use television (ILI-LAR 49.85%, ILA-MAR 38.77%), social networks (ILI-LAR 26.40%, ILA-MAR 20.51%) and newspapers (ILI-LAR 9.22%, ILA-MAR 18.87%).

Figure 6c compared the level of trust between ILI and ILA about the information received by several actors regarding COVID-19. In detail, around 39% of ILI and 51% of ILA expressed either few or none trust in mass media, whereas both ILI and ILA demonstrated a high level of trust in the information received by National Government (almost 80%), Italian Civil Protection Department (almost 80%) and government at local level (almost 65%). However, it has to be noted that both ILI and ILA have a lower level of trust in general practitioner (almost 60%) than in the above-cited institutions about information related to this outbreak. This might be a starting point for further insights, inasmuch as there is the need to understand the reason beyond this discrepancy, as well as to give the appropriate level of confidence to the experts in the field. 

To whom regard the difference between the trust related to the information received by relatives and friends, ILA resulted more confident about the information received from them (around 53% expressed much and very much level of trust) than ILI (almost 31%). Probably, they trust more closed relatives and friends because ILA live the Italian emergency “from outside”, thus looking for confirmation about the news collected by other sources. 

### 4.5. Perceived Efficacy of Mitigation Measures by the Italian Government

**Figure 7:**
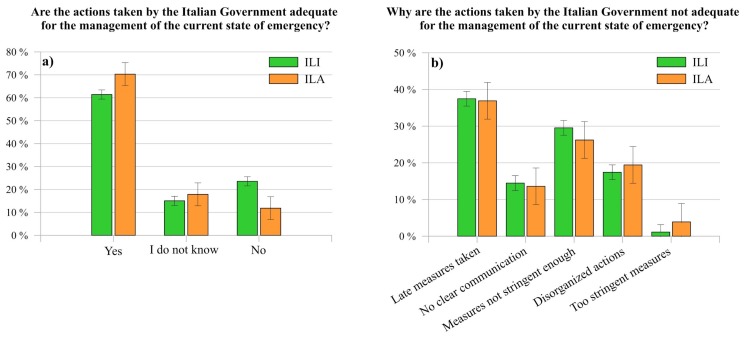
Comparison between ILI and ILA about the adequacy of the actions taken by the Italian Government (**a**) for the management of the Italian COVID-19 state of emergency, and (**b**) about the reasons of the respondents who do not agree with them.

Figure 7a shows the results associated to the question “Are the actions taken by the Italian Government adequate for the management of the current state of emergency?”. In general, despite their severity, both ILI and ILA expressed a positive feeling related to them (ILI 61.41%, ILA 70.30%). As regard to those who highlighted the inadequacy of the adopted mitigation measures (23.55% ILI and 11.83% ILA), Figure 7b reports the reasons of their negative evaluation. Specifically, around 37% of both clusters stated that actions were applied belatedly, whereas 29.53% of ILI and 26.21% of ILA affirmed that they were not enough stringent. Moreover, less than 20% of respondents defined the measures disorganized, highlighting a lack of clear communication (around 14% of both ILI and ILA).

Finally, only a negligible part of them (ILI 1.11%, ILA 3.88%) considered the adopted measures too stringent. Thus, the findings indicate a widespread acceptance of the government measures by the interviewees, even though they limit the freedom of movement and interaction of individuals and, therefore, lead to a full and sudden change in daily lives routine. This can also be related to the high level of confidence about information received from these institutions, as already highlighted in the previous paragraph. Specifically, as previously reported [64], severe and strict security measures are more likely to be accepted in a situation of shared uncertainty and fear (see Figure 4). 

Regarding the perception of ILA about the comparison between the actions adopted by the Italian Government compared to ones adopted (or not) by the Countries where they live, most of respondents (71.46%) affirmed to consider either better or much better the Italian actions, whereas 19.02% considered them equal to ones adopted by the Country where they live. Finally, just less than one in ten Italian living abroad (9.52%) stated that Italian mitigation measures were either worse or much worse than ones adopted in their living country. Such result might be influenced by the fact that Italy was one of the first countries, after China, which adopted draconian measures to limit the spread of SARS-CoV-2. As a matter of fact, the result of the comparison between Italian actions and the ones adopted in the countries where ILA live is in line with their perception about the adequateness of Italian actions for the management of the current state of emergency (Figure 7). 

### 4.6. The Role of Information Sources on the Health Risk Perception of ILI-MAR and ILI-LAR

As already highlighted, some differences emerge between answers provided by ILI-MAR and ILI-LAR. In order to better investigate these diversities, a further analysis on cross data considering the answers of participants according to their different way to collect information has been carried out. Since most of the ILI uses television, social networks, and newspapers to follow the situation about the current outbreak, the present paragraph compares in-deep some answers of ILI-MAR and ILI-LAR according to the main sources of information about COVID-19.

Generally, it is possible to note a difference between two groups of people according to the main information source: ILI who read newspapers and ILI who collect information from social networks and television. In deep, discrepancies of feelings, level of knowledge and level of acceptance related to the Italian government mitigation measures are more evident between ILI-MAR who use newspapers and ILI-LAR who collect information through social networks and television.

**Figure 8:**
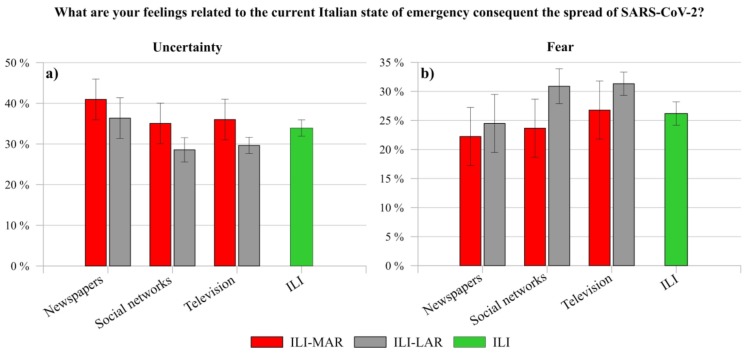
Cross data comparison between ILI-MAR and ILI-LAR about the experienced feelings of (**a**) uncertainty and (**b**) fear according to the main source of information of respondents.

Specifically, uncertainty is on average higher in people who collect information through newspapers rather than in ones who mainly use social networks and television (Figure 8a). On the contrary, the latter result more fearful than the former (Figure 8b). This reflects the same result obtained by comparing ILI-MAR (more uncertain) and ILI-LAR (less uncertain) (see Section 4.2), but with a new perspective related to the main used source of information.

**Figure 9:**
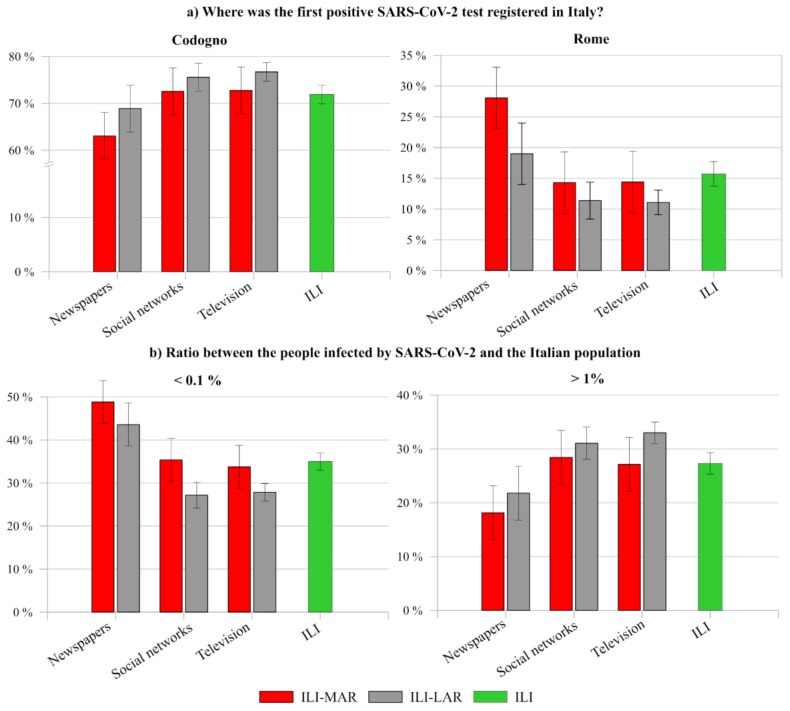
Cross data comparison between ILI-MAR and ILI-LAR related to their level of knowledge about (**a**) the place of the first positive SARS-CoV-2 test in Italy, and (**b**) the ratio between people infected by SARS-CoV-2 and the Italian population, according to the main source of information of respondents.

By continuing the comparison, a similar situation emerges also regarding the level of knowledge of the respondents, in particular about the place (Figure 9a) of the first positive SARS-CoV-2 registered in Italy. First at all, although a general high percentage of wrong answers among interviewees still remains, slight variations arise compared to the national trend (see Section 4.3). On the one hand, people who collect information through newspapers, in particular ILI-MAR, result more aware about both the correct place and date. On the other hand, respondents who mainly use social networks and television, in particular ILI-LAR, result with the higher percentage of wrong answers. The same trend can be observed according to the ratio between the people infected by SARS-CoV-2 in Italy and the whole population (Figure 9b).

**Figure 10:**
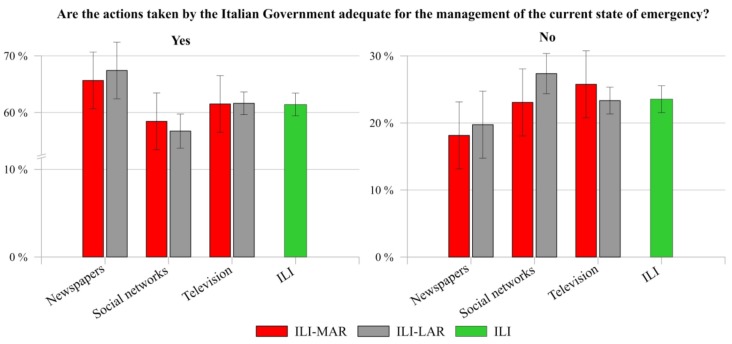
Cross data comparison between ILI-MAR and ILI-LAR related to their acceptance about the actions taken by Italian Government in the state of emergency, according to the main source of information of respondents.

The last remarkable result regards the acceptance of the strict actions applied by the Italian Government (Figure 10). In this case, the discrepancy is slight visible among ILI reading newspapers, who accept the strategies, and ILI mainly using social networks, who disagree with them.

Summing up, it is possible to gather that the above-discussed differences associated to feelings, level of knowledge and level of acceptance of the mitigation measures is exacerbated by the source of information used by ILI. As the matter of fact, people who mainly collect information through *newspapers*, in particular ILI-MAR, result the ones with the higher level of knowledge. Probably, their awareness about the complex and hazardous situation that Italy is currently facing, leads them to be more uncertain and to accept more the imposed mitigation measures, such as lockdown and quarantine. On the contrary, it would seem that the prevalent use of television and social networks, in particular among ILI-LAR, contributes to elicit a prevalent state of fear in individuals. It might be related to their lower level of knowledge and, consequently, their lesser inclination to accept the strict mitigation measures.

As regard to both the level of knowledge and feelings, it is worthy to report that many fake and misleading news were reported by all the mass media, specifically social networks. The effect of these news promotes a wrong perception of risk [65]. The Italian government have become conscious of this issue. Thus, on April 4, 2020 [66], the Italian Under-Secretary with Delegation to Information and Publishing, Andrea Martella, set up a task force to fight against the spread of fake news. However, the adoption of measures to contrast such spread is still debated since it could limit the freedom of expression guaranteed by article 11 of the Charter of Fundamental Rights of the European Union [67], as well as article 21 of the Italian Constitution [68].

### 4.7. Preliminary Insights on the Perceived Future Effects on Interpersonal Distances

Since social distancing is the first defense against the spread of COVID-19 outbreak, one of the aspects investigated in this work is the foresee variation of interpersonal distances after the end of the Italian state of emergency. To the question “In your opinion, at the end of the state of emergency issued by the Italian Government, what will the following interpersonal distances be like?”, respondents generally expect some changes in the future interpersonal distances (intimate, personal, social and public). Moreover, the general trend does not show consistent differences between ILI and ILA perceptions.

**Figure 11:**
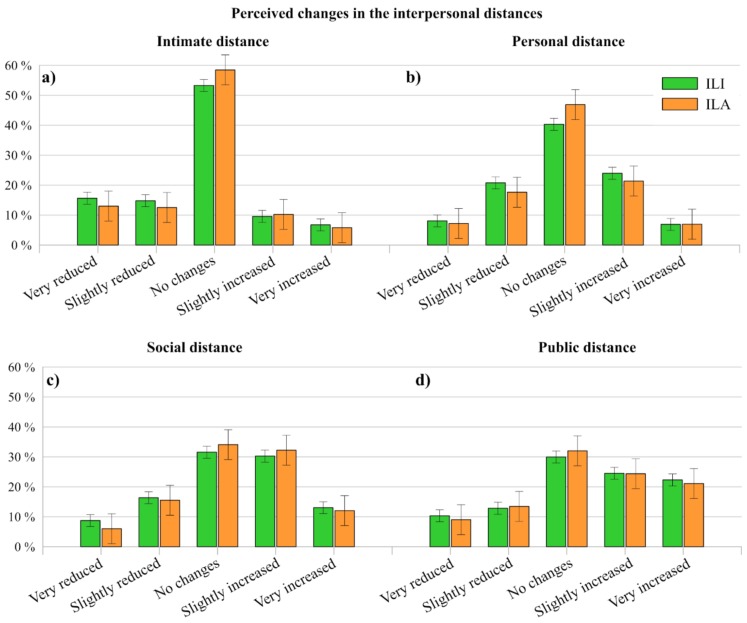
Comparison between the foreseen variations of ILI and ILA interpersonal distances: (**a**) intimate, (**b**) personal, (**c**) social, and (**d**) public.

Looking at Figure 11, it is possible to note that some first differences emerge between two sub-groups, that are intimate-personal distances and social-public distances. In fact, at first sight, intimate and personal distances do not seem to be expected to change then before the sanitarian emergency state, while the perception about social and public distances appears a little different.

Getting into detail, intimate distance for both ILI and ILA (Figure 11a) is perceived to be almost the same for more than the 50% of respondents. In fact, 53.30% of ILI and 58.47% of ILA stated that no changes are expected for the intimate distance and 40.31% of ILI and 46.87% of ILA for the personal distance (Figure 11b). Regarding the foreseen variations expressed by respondents, intimate distance is expected to be more reduced instead of increased. To whom concern personal distance, an equal trend is registered between those who stated that distances will be either reduced or increased. Regarding social (Figure 11c) and public distances (Figure 11d), results show an expected an increase of these lengths after the end of the Italian emergency (for social distance: ILI 43.31%, ILA 44.31%; for personal distance: ILI 46.84%, ILI 45.47%). Thus, since Hall [56] associates the interpersonal distances to cultural aspects, it seems that the spread of the virus in Italy is going to cause a variation of Italian cultural behaviors.

## 5. Conclusions

This work provided preliminary insights, which might support decision makers to face future sanitary emergencies, by investigating the public perception of health risk of COVID-19 in Italy. To this purpose, a questionnaire was designed and, consequently, administered within the period of lockdown and quarantine in Italy to almost 9000 Italians living in Italy (ILI) and abroad (ILA). The investigated aspects regarded the level of acceptance and the level of knowledge, as well as some effects of this public health emergency to the feelings, concerns, and behaviors of ILI and ILA.

Notably, it is worthy to report that the higher the level of knowledge regarding the Italian situation, the higher the uncertainty perceived by the respondents, as well as the level of acceptance of the mitigation measures. Probably, the better awareness about this complex and hazardous situation leads people to be more uncertain and to accept more strict mitigation measures, such as lockdown and quarantine.

Moreover, some differences emerged between the responses of Italians living in more affected regions (ILI-MAR) and Italians living in less affected regions (ILI-LAR). As reported within the study, another aspect influencing the perception of the respondents regards the main mass media (such as television, social networks, and newspapers) adopted to collect information about the spread of SARS-CoV-2 in Italy. Generally, interviewees who used newspapers as a main source of information, in particular ILI-MAR, were more aware about the COVID-19 pandemic in Italy. On the contrary, it would seem that the prevalent use of television and social networks, in particular among ILI-LAR, contributes to a lower level of knowledge and to elicit a prevalent state of fear in individuals.

This study confirms the key role of mass media on the risk perception, level of knowledge regarding the emergency situation, the level of acceptance of mitigation measures, and the perceived feelings by the Italian population. Consequently, the diffusion of fake news and misleading information may result in an incorrect perception of risk. For this reason, governments should implement measures to face with this issue as the Italian Government has already began to do. However, it is a tricky topic since these measures can limit the freedom of expression guaranteed by the national and international legislations.

Finally, first insights about the perceived future effects of Italian population caused by the outbreak were given. In detail, analyzing data regarding the variation of interpersonal distances, it seems that the spread of the virus in Italy could cause a change of Italian cultural behaviors, e.g., varying such distances. However, there is the need to further investigate such potential variations, inasmuch as there is no evidence that perceived changes in patterns of behaviors will be permanent.

## Figures and Tables

**Table 1:** Participation rates and distributions of the basic demographic information of the studied population.

Variable	ILI		ILA	
	N	%	N	%
**Total n**	8282	100	431	100
*Gender*				
Male	3326	40.20	164	38.10
Female	4956	59.80	267	61.90
*Age*				
<25	1325	16.00	77	17.90
26–35	2507	30.30	214	49.70
36–50	2398	29.00	97	22.50
51–65	1595	19.30	33	7.70
>65	457	5.50	10	2.30
*Level of education*				
<High school	552	6.70	8	1.90
High school	3011	36.40	99	23.00
University/post-lauream	4719	57.00	324	75.20
*Job status*				
Employee	3967	47.90	255	59.20
Autonomous/entrepreneur	1494	18.00	49	11.40
Student	1364	16.50	89	20.60
Retiree	564	6.80	12	2.80
Unemployed	893	10.80	26	6.00

**Table 2:** Considered clusters, sub-clusters, sample size, confidence levels and intervals.

Cluster Sample	Sub-Cluster Sample	Sample Size	Confidence Level	Confidence Interval
ILI-MAR	//	2453	95%	±2%
	Information mainly from newspapers	463	95%	±5%
	Information mainly from social networks	503	95%	±5%
	Information mainly from television	951	99%	±5%
ILI-LAR	//	5829	99%	±2%
	Information mainly from newspapers	537	95%	±5%
	Information mainly from social networks	1539	95%	±5%
	Information mainly from television	2906	95%	±3%
ILI^1^	//	8282	95%	±2%
ILA	//	431	95%	±5%

^1^ The results of ILI are the weight mean of the results of ILI-MAR and ILI-LAR.
